# Antia, a Natural Antioxidant Product, Attenuates Cognitive Dysfunction in Streptozotocin-Induced Mouse Model of Sporadic Alzheimer's Disease by Targeting the Amyloidogenic, Inflammatory, Autophagy, and Oxidative Stress Pathways

**DOI:** 10.1155/2020/4386562

**Published:** 2020-06-17

**Authors:** Nesrine S. El Sayed, Mamdooh H. Ghoneum

**Affiliations:** ^1^Department of Pharmacology and Toxicology, Faculty of Pharmacy, Cairo University, Cairo, Egypt; ^2^Department of Surgery, Charles Drew University of Medicine and Science, Los Angeles, California, USA

## Abstract

**Background:**

Many neurodegenerative diseases such as Alzheimer's disease are associated with oxidative stress. Therefore, antioxidant therapy has been suggested for the prevention and treatment of neurodegenerative diseases.

**Objective:**

We investigated the ability of the antioxidant Antia to exert a protective effect against sporadic Alzheimer's disease (SAD) induced in mice. Antia is a natural product that is extracted from the edible yamabushitake mushroom, the gotsukora and kothala himbutu plants, diosgenin (an extract from wild yam tubers), and amla (Indian gooseberry) after treatment with MRN-100.

**Methods:**

Single intracerebroventricular (ICV) injection of streptozotocin (STZ) (3 mg/kg) was used for induction of SAD in mice. Antia was injected intraperitoneally (i.p.) in 3 doses (25, 50, and 100 mg/kg/day) for 21 days. Neurobehavioral tests were conducted within 24 h after the last day of injection. Afterwards, mice were sacrificed and their hippocampi were rapidly excised, weighed, and homogenized to be used for measuring biochemical parameters.

**Results:**

Treatment with Antia significantly improved mice performance in the Morris water maze. In addition, biochemical analysis showed that Antia exerted a protective effect for several compounds, including GSH, MDA, NF-*κ*B, IL-6, TNF-*α*, and amyloid *β*. Further studies with western blot showed the protective effect of Antia for the JAK2/STAT3 pathway.

**Conclusions:**

Antia exerts a significant protection against cognitive dysfunction induced by ICV-STZ injection. This effect is achieved through targeting of the amyloidogenic, inflammatory, and oxidative stress pathways. The JAK2/STAT3 pathway plays a protective role for neuroinflammatory and neurodegenerative diseases such as SAD.

## 1. Introduction

Age-related neurodegenerative diseases like Alzheimer's disease (AD) are on the rise. AD is a disorder characterized by progressive deterioration of cognition and memory, and it accounts for 60-80% of all dementia cases [1]. Among the elderly, the most common type of AD is sporadic Alzheimer's disease (SAD), a type that involves the central nervous system's progressive degeneration [2]. Several pathways have been examined as possible targets for SAD, including the oxidative stress, amyloidogenic, inflammatory, and autophagy pathways.

One of the earliest changes in AD brains is the appearance of oxidative stress markers, which precedes the accumulation of neurofibrillary tangles and visible amyloid deposits [3]. Oxidative stress is implicated in many disorders like chronic inflammation, AD, and Parkinson's disease [4]. The brain's neurons have high rates of energy production and oxygen consumption, making them extremely sensitive to excessive generation of reactive oxygen species (ROS) and oxidative damage [5].

In AD brains, normally solid amyloid *β* (A*β*) and tau proteins assemble into amyloid-like filaments called plaques and tangles. The manner by which A*β* accumulates in the central nervous system and triggers cell disease is currently unresolved, but a suggested mechanism by which A*β* may damage neurons and cause neuronal death includes ROS generation during A*β* self-aggregation. When this process was observed in vitro on neuron membranes, it ultimately led to mitochondrial impairment, excessive calcium influx, and synaptic membrane depolarization [6, 7].

Neurodegenerative diseases like AD are also accompanied by neuroinflammation. The inflammatory response of neurons has been linked with the transcription factor NF-*κ*B. In normal conditions, NF-*κ*B forms an inactive cytoplasmic complex with I*κ*B*α*, its inhibitor. When NF-*κ*B is stimulated, however, it can induce the transcription of inflammatory target genes such as interleukin-1*β* (IL-1*β*), interleukin-6 (IL-6), tumor necrosis factor-*α* (TNF-*α*), and cyclooxygenase-2 (COX-2). In addition, neuroinflammation has been linked with autophagy in neurodegenerative diseases. Neuroinflammation can result in a deficit of autophagy that exacerbates neurodegeneration and, conversely, a disruption of autophagy during pathological conditions can initiate or intensify neuroinflammation [8]. In human AD and in mouse models of AD, decreased autophagy has been observed and found to contribute to pathological build-up of tau aggregates [9]. Autophagy is known to be regulated by mTOR, rapamycin's mammalian target, and mTOR inhibition has been found to prevent neuroinflammation in a mouse model of cerebral palsy [10]. Moreover, in a study of rat cortices subjected to ischemic brain injury, it has been shown that GSK-3*β* inhibition suppresses neuroinflammation through autophagy activation [11].

Pharmacological management of AD has been limited to date. In 2007, long-term use of nonsteroidal anti-inflammatory drugs (NSAIDs) was considered to be linked with a reduced probability of developing AD [12]. NSAIDs were also indicated by evidence to potentially reduce amyloid-plaque-related inflammation, but high adverse events caused a suspension of trials [13]. AD risk has not been found to decrease with any medications or supplements [13], and unfortunately, current treatments for AD that are FDA-approved offer only symptomatic relief and are not able to cure or delay the disease [1].

Recently, antioxidants have received increased attention in preventing the onset of AD by reducing oxidative stress insult [14, 15]. Furthermore, there has been an acceleration in the search for and use of drugs and dietary supplements from plants, due in part to the health benefits that have been found in phytochemicals whose uses have been documented in traditional medicine [16]. Components of the traditional Chinese medicinal mushroom called yamabushitake promote nerve growth factor synthesis in cultured astrocytes [17, 18] as well as improving mild cognitive impairment in humans [19]. The gotsukora plant has traditionally been used for dementia and memory improvement [20, 21], and its extracts have been shown to improve memory retention in rodents [22], to alter amyloid beta pathology in the hippocampi of a mouse model of AD, and to modulate the oxidative stress response involved in AD-related neurodegeneration [23]. Diosgenin, a plant-derived steroidal sapogenin, has been shown to exert anticancer effects [24], improve aging-related cognitive deficits [25], and relieve diabetic neuropathy [26]. Recently, it was proven that diosgenin improves memory function and reduces axonal degeneration in AD mouse models [20, 27]. Amla (Emblica officinalis), the Indian gooseberry, has been shown to exert diverse neuroprotective pharmacodynamic actions [28]; to have potent radical scavenging effects [29]; to have a high degree of neuroprotective potential in a panel of bioassays that targeted protein glycation, carbonyl stress, acetylcholinesterase inhibition, oxidative stress, A*β* fibrillation, and neuroinflammation [30]; and to improve the acetylcholinesterase activity, brain antioxidant enzymes, and cognitive functions in a rat model of AD [31]. Finally, kothala himbutu (Salacia reticulata) has been shown to protect against deleterious cognitive changes in young streptozotocin-induced diabetic rats [32] and against mercury toxicity in mice hippocampi [33].

In this study, we examine the cogno-protective effects of an antioxidant product called Antia whose components include yamabushitake, gotsukora, diosgenin, amla, and kothala himbutu. These components are treated together with the hydroferrate fluid MRN-100 to generate Antia. Long-term administration of MRN-100 revealed its protective effect against age-associated oxidative stress [34] and against oxidative damage in human leukemia cells and in endothelial cells [35]. Recent studies on Antia have shown its ability to reverse mitochondrial dysfunction caused by oxidative stress in human peripheral blood lymphocytes [36]. In light of the above-mentioned neuroprotective effects of Antia's plant components, we hypothesized that Antia would have beneficial effects on the pathways relevant to AD, namely, the oxidative stress, amyloidogenic, inflammatory, and autophagy pathways. We studied the effect of Antia on mice induced with SAD via intracerebroventricular (ICV) injection of streptozotocin (STZ); this is a well-established animal model of SAD based on brain resistance to insulin [37] and imitates the age-related pathology of SAD in humans such as memory impairment, oxidative stress, neuroinflammation, and neurodegeneration [38]. Here, we present behavioral, biochemical, and western blot experiments in support of our hypothesis.

## 2. Materials and Methods

### 2.1. Animals

Adult male albino mice 25-30 g in weight were provided by the animal facility of the Faculty of Pharmacy, Cairo University, Egypt. For one week before conducting the study, the mice were allowed to acclimate. Mice were housed in a controlled environment with constant temperature (25 ± 2°*C*), relative humidity of 60 ± 10%, and a 12/12 h light/dark cycle. Standard chow diet and water were allowed ad libitum. Every effort was made to minimize mice suffering and to reduce the mice number used. This study was approved by the Ethics Committee for Animal Experimentation and complied with the recommendations of the National Institutes of Health Guide for Care and Use of Laboratory Animals (2011).

### 2.2. Chemicals

STZ was purchased from Sigma-Aldrich Co. (St Louis, MO, USA). STZ was dissolved in saline solution (0.9% NaCl) and injected ICV at a volume of 10 *μ*L by the freehand method. Antia was dissolved in saline solution in three doses: 25 mg/kg equivalent to the adult dose (4 tablets/day), 50 mg/kg, and 100 mg/kg. It was then administered intraperitoneally (i.p.) at a volume of 0.1 mL/20 g-mouse. Each day, fresh drug solutions were prepared. Equivalent volumes and administration routes were used for the control group's saline injections. All other chemicals were of the highest analytical grade.

### 2.3. Antia

Antia is a natural compound derived from a variety of mushrooms and plants, including the edible yamabushitake mushroom, the gotsukora and kothala himbutu plants, diosgenin (an extract from the tubers of Dioscorea wild yam), and amla (Indian gooseberry). The ingredients are treated with an iron-based fluid called MRN-100. MRN-100 is made from phytosin and is an iron-based compound derived from bivalent and trivalent ferrates (hydroferrate fluid). The exact chemical composition of Antia is still under active investigation. Antia was provided by ACM Co., Ltd, Japan. Antia was prepared in distilled water (DW) with the concentration of MRN-100 at about 2 × 10^−12^ mol/L.

### 2.4. Induction of SAD

SAD was induced by ICV injection of STZ (3 mg/kg) into the lateral ventricle of mice according to the freehand procedure [39] and as updated by Warnock [40] to avoid the probability of cerebral vein penetration. Anesthetization of mice was carried out with thiopental (5 mg/kg, i.p.). Their heads were stabilized with downward pressure above the ears, and a needle was directly inserted through the skin and skull into the lateral ventricle. The bregma was located by visualizing an equilateral triangle between the center of the skull and the eyes, allowing the needle to be inserted approximately 1 mm lateral to this point. Normal behavior was observed one minute after injection.

### 2.5. Experimental Design

**Figure 1:**
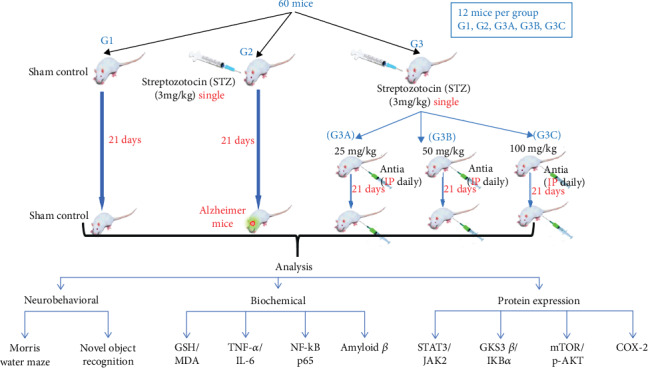
Experimental design.

The experimental design is illustrated in Figure 1. Mice were divided randomly into five groups containing 12 animals each. Group I (sham control): mice received ICV injection once and intraperitoneal (i.p.) saline injection for 21 consecutive days and served as the sham control group. Group II (STZ): mice received STZ (3 mg/kg, ICV) once and served as a model for SAD [41]. Group III (STZ+Antia 1): mice received STZ (3 mg/kg, ICV) followed by Antia (25 mg/kg, i.p.) after five hours and then every day for 21 consecutive days. Group IV (STZ+Antia 2): mice received STZ (3 mg/kg, ICV) followed by Antia (50 mg/kg, i.p.) after five hours and then every day for 21 consecutive days. Group V (STZ+Antia 3): mice received STZ (3 mg/kg, ICV) followed by Antia (100 mg/kg, i.p.) after five hours and then every day for 21 consecutive days. 24 h after the end of treatments, neurobehavioral tests were carried out, including object recognition and Morris water maze (MWM) tests, sequentially arranged from the least to most stressful. Testing was performed during animals' light cycle under top illumination to minimize circadian variability. No mortality was observed among all the animals in different groups during the experimental period of 21 days.

### 2.6. Acute Toxicity Study

The acute toxicity of *Antia* was evaluated in mice using the up and down procedure according to the Organization for Economic Cooperation and Development (OECD), guideline no. 423, 2001 [42].

Mice received Antia starting at a dose of 2 g/kg. The animals were observed for toxic symptoms continuously for 24 hours and then maintained for additional 20 days with daily observations.

### 2.7. Behavioral Assessments

#### 2.7.1. Object Recognition Test

Long-term memory and cognition were assessed via the object recognition test [43]. In this study, the performed tests took place over three consecutive days. On day one (the habituation phase), each mouse was individually placed into a wooden box of dimensions 30 × 30 × 30 cm^3^ for 30 min in order to adapt to the surrounding environment. Day two was designed for familiarization or training, where two wooden cubes identical in shape, color, and size were placed in opposite corners of the box, 2 cm from the walls. Each mouse was placed in the middle of the box and was left to explore these two objects for 10 min. On day three, testing took place. A novel object that was different from the identical cubes in shape, size, and color was used to replace one of the two identical cubes. Each mouse was exposed again to these two objects for 5 min. 70% ethanol was used to clean objects between animal experiments to ensure that odor cues did not guide behavior. All locations and objects were adjusted to decrease biases due to inclinations for particular objects or locations. Mice were always placed in the box confronting the same wall, and mice were unable to physically move the objects. The animals' behavior was video-recorded and the following parameters were calculated:

1. Discrimination index: temporal difference between exploring the novel object and the familiar object divided by the total time used to explore both objects. The measurement varies between -1 and +1, where a negative (positive) score indicates that more (less) time was used to explore the familiar object, and a score of zero indicates no preference
2. Preference index: time spent by the animal exploring the novel object as a percentage of the total time exploring both objects

#### 2.7.2. Morris Water Maze Test

Spatial learning and memory was assessed via the Morris water maze (MWM) test [44]. The maze consisted of stainless-steel circular tanks (210 cm in diameter, 51 cm high) filled with water (25 ± 2°*C*, 35 cm deep) and divided into four quadrants. A submerged black platform (10 cm width, 28 cm height) was placed in a target quadrant 2 cm below the surface of the water. This platform remained at a consistent position during the time of training and the test. A nontoxic purple dye was added to the water to obscure any visible evidence of the platform's position. Trials for memory acquisition (120 s/trial) were performed 2x/day over four consecutive days, with a period between trials of at least 15 min. For each acquisition trial, mice were left free to locate the hidden platform in the target quadrant. If a mouse located the platform, it was allowed to rest there for 20 s, while if a mouse failed to locate the platform within 120 s, it was gently guided to the platform and allowed to rest there for 20 s. The mean escape latency was measured as the time taken by each mouse to locate the hidden platform and was used as an index of learning or acquisition. On day five, the mice were given a probe-trial session. The platform was taken out of the pool and each mouse was given 60 s to probe the pool. The time each mouse spent in the target quadrant of the previously placed platform was measured as an indicator of memory or retrieval.

### 2.8. Brain Processing

After behavioral testing, cervical dislocation was used to euthanize mice and brains were rapidly dissected and washed with ice-cold saline. The hippocampi (*n* = 6) were excised from each brain on an ice-cold glass plate. The hippocampus was homogenized in ice-cold saline to prepare 10% homogenates. These were split into several aliquots and stored at -80°C. The other hippocampus was stored at -80°C to be used for western blot analysis.

### 2.9. Biochemical Measurements

#### 2.9.1. Determination of Oxidative Stress Biomarkers

Hippocampal lipid peroxidation was estimated by measuring malondialdehyde (MDA) levels. MDA was determined by measuring the thiobarbituric acid reactive substances as described in [45]. Brain glutathione (GSH) content was measured spectrophotometrically using Ellman's reagent as described in [46]. The results are expressed as mmol/mg protein.

#### 2.9.2. Determination of Inflammatory Biomarkers and Amyloid *β*1-42

Hippocampal TNF-*α*, IL-6 levels, NF-*κ*B p65, and *β*-amyloid_1-42_ were measured using mouse ELISA kits purchased from RayBiotech Inc. (Norcross, GA, USA) and R&D Systems Inc. (Minneapolis, USA), respectively. The procedures were performed according to the manufacturers' instructions. Results are presented as pg/mg protein for TNF-*α*, IL-6, NF-*κ*B p65, and *β*-amyloid_1-42_.

#### 2.9.3. Western Blot Analysis

Protein solutions were extracted from brain tissues, after which equal protein amounts (20–30 *μ*g of total protein) were separated by SDS-PAGE (10% acrylamide gel) and transferred to polyvinylidene difluoride membranes (Pierce, Rockford, IL, USA) with a Bio-Rad Trans-Blot system. Western blot immunodetection was performed by incubating membranes for 1 h at room temperature with blocking solution comprised of 20 mM Tris-Cl, pH 7.5, 150 mM NaCl, 0.1% Tween 20, and 3% bovine serum albumin. Membranes were incubated overnight at 4°C with one of the following primary antibodies: P-JAK2 (Tyr 1007/1008), P-STAT3 (Tyr 705), I*κ*B*α*, GSK-3*β*, mTOR, COX-2, or *β*-actin, obtained from Thermo Fisher Scientific Inc. (Rockford, IL, USA). Peroxidase-labelled secondary antibodies were added after washing, and the membranes were incubated at room temperature for 1 h. ChemiDoc™ imaging system with Image Lab™ software version 5.1 (Bio-Rad Laboratories Inc., Hercules, CA, USA) was used to analyze the band intensity. Results are presented in arbitrary units after normalization to levels of the *β*-actin protein.

#### 2.9.4. Determination of Protein Content

The method of Bradford was used to measure protein content. All results are expressed as tissue concentration per mg protein.

### 2.10. Statistical Analysis

The presented data are mean ± S.E. One-way analysis of variance (ANOVA) followed by the Tukey-Kramer multiple comparison test was used in data analysis. GraphPad Prism software (version 6; GraphPad Software, Inc., San Diego, CA, USA) was used for statistical analysis and the creation of graphical presentations. Significance levels of all statistical tests are set at *p* < 0.05.

## 3. Results

The acute toxicities of the tested *Antia* were studied, and the results showed no general behavior changes, toxicity, and mortality in test animals up to the dose level of 2 g/kg during a 24 h period, thus indicating that this substance had no toxicity.

**Figure 2:**
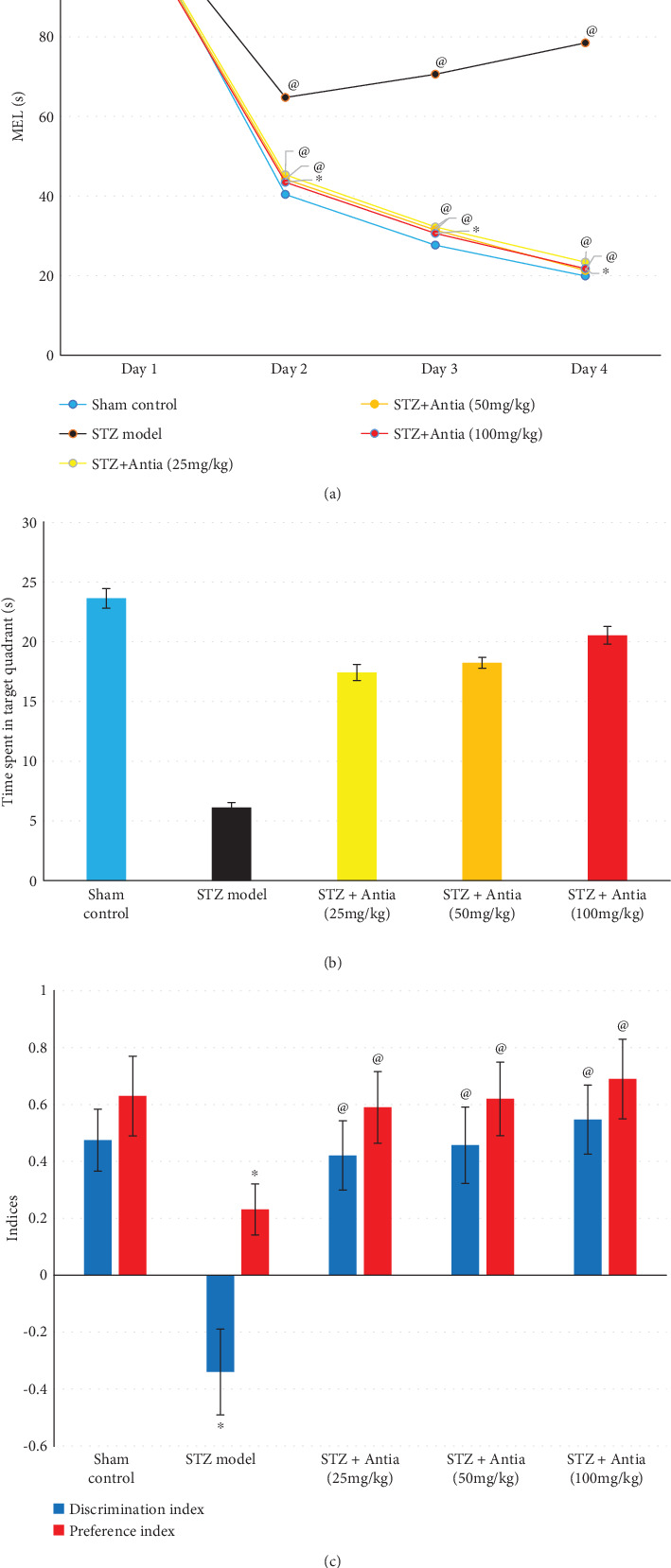
(a) Effect of Antia on mean escape latency (MEL) in Morris water maze, (b) effect of Antia on time spent in target quadrant in Morris water maze, and (c) effect of Antia on cognitive function in the novel object recognition test for ICV-STZ-injected mice. ^∗^Significantly different from the sham control group at *p* < 0.05. ^@^Significantly different from the ICV-STZ group at *p* < 0.05.

The effects of Antia on the behavioral and biochemical functions of ICV-STZ-treated mice were measured with neurobehavioral tests and biochemical analysis of the hippocampal content. The effects of STZ and Antia (25, 50, and 100 mg/kg) on neurobehavioral tests were carried out within 24 h after the last day of Antia injection. The Morris water maze was used to examine the possible protective effect of Antia treatment on ICV-STZ-injected mice. As illustrated in Figure 2(a) for the mean escape latency (MEL), mice in different groups took different times to escape on day 2. STZ-treated mice took 1.63 times as long to escape on day 2 as compared to sham control mice. Treatment of STZ-injected mice with Antia, however, took only 1.08 times as long as sham control mice on day 2. These results were further confirmed in the subsequent days 3 and 4. The study of the effect of Antia on the time mice spent in the target quadrant of the Morris water maze (Figure 2(b)) showed that STZ-treated mice spent only 25.4% of the time in the quadrant in comparison with sham control mice, while treatment of STZ-injected mice with 25, 50, and 100 mg/kg of Antia spent 72.5%, 75.8%, and 85.4% of the time, respectively, in comparison with sham control mice.

The effect of STZ and Antia was further examined through the discrimination and preference indices of the novel object recognition test. The discrimination index was decreased in STZ-induced SAD mice when compared to the sham control group, but it was significantly increased after Antia administration (25, 50, and 100 mg/kg) as compared to the STZ group in a dose-dependent manner. In addition, the time spent exploring the novel object was lower in ICV-STZ-injected mice by 63% compared to the sham control group, reflecting a lower preference index. Antia administration (25, 50, and 100 mg/kg) normalized the preference index, indicating that Antia-treated mice preferred the novel object over the familiar object in a dose-dependent manner (Figure 2(c)).

**Figure 3:**
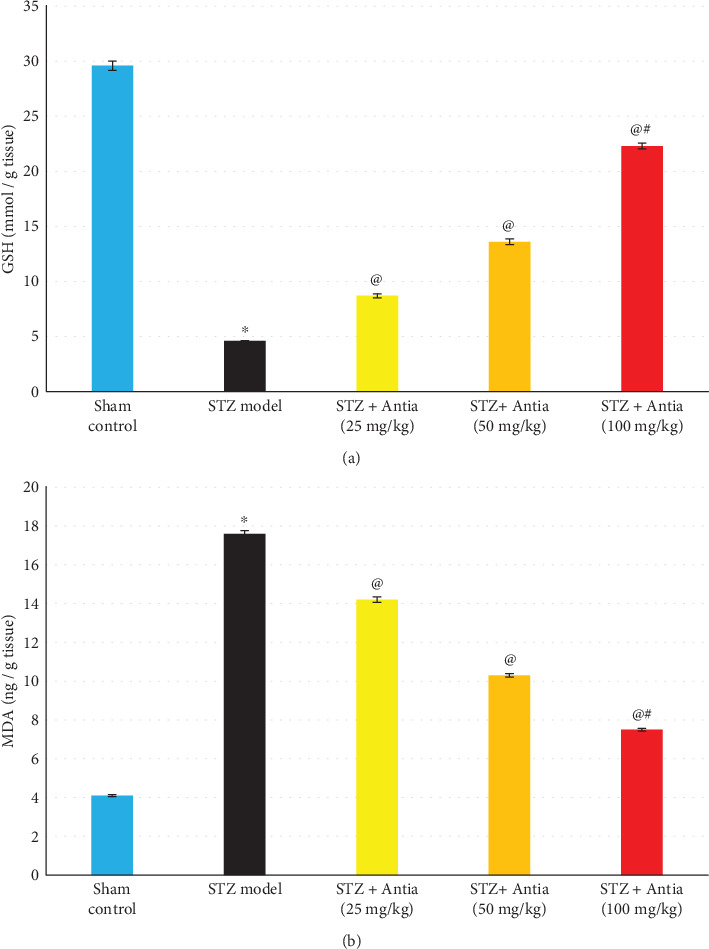
(a, b) Effect of Antia on GSH and MDA hippocampal content in ICV-STZ-injected mice. ^∗^Significantly different from the sham control group at *p* < 0.05. ^@^Significantly different from the ICV-STZ group at *p* < 0.05. ^#^Significantly different from Antia (25 mg/kg) at *p* < 0.05. ^$^Significantly different from Antia (50 mg/kg) at *p* < 0.05.

Several biochemical analyses of the hippocampal content in ICV-STZ-treated mice were conducted in order to examine the ability of Antia to attenuate the amyloidogenic, inflammatory, autophagy, and oxidative stress pathways. Measurements of the protective effect of Antia treatments on the levels of malondialdehyde (MDA) and glutathione (GSH) hippocampal content were carried out. Results in Figure 3(a) show that STZ-treated mice had a GSH level that was 15.5% of the GSH level of sham control mice. On the other hand, treatment of STZ-injected mice with Antia showed an elevation in the GSH content in a dose-dependent manner that maximized at 78.7% of the control GSH level for 100 mg/kg Antia treatment. Results of the levels of MDA hippocampal content show significantly higher levels of MDA in ICV-STZ-injected mice as compared with sham control mice by a factor of 4.3-fold. On the other hand, Alzheimer's mice with Antia showed an elevation in the MDA content of only 3.5-fold, 2.5-fold, and 1.8-fold for mice receiving Antia at doses of 25, 50, and 100 mg/kg, respectively (Figure 3(b)).

**Figure 4:**
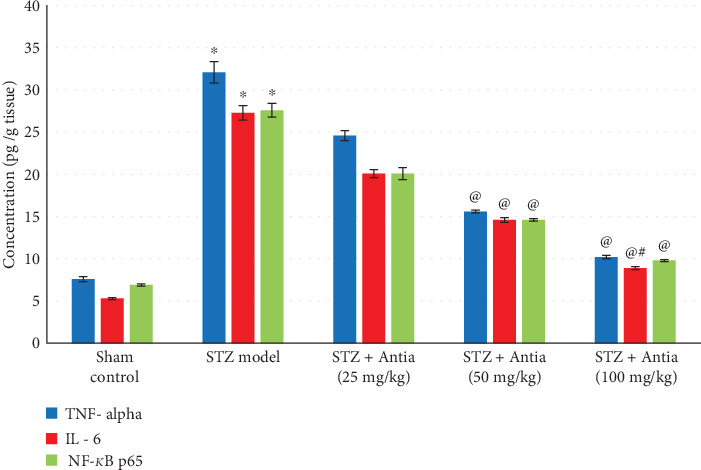
Effect of Antia on TNF-*α*, IL-6, and NF-*κ*B p65 hippocampal content in ICV-STZ-injected mice. ^∗^Significantly different from the sham control group at *p* < 0.05. ^@^Significantly different from the ICV-STZ group at *p* < 0.05. ^#^Significantly different from Antia (25 mg/kg) at *p* < 0.05. ^$^Significantly different from Antia (50 mg/kg) at *p* < 0.05.

The effect of ICV-STZ injection on the hippocampal content of anti-inflammatory cytokines was also examined in the presence and absence of Antia treatment. Two cytokines were examined: TNF-*α* and IL-6. Results in Figure 4 show that STZ model mice exhibited a significant increase in TNF-*α* and IL-6 cytokine expression as compared with sham control, but Antia treatment suppressed this induction in a dose-dependent fashion, reaching the control's level at 100 mg/kg. A similar trend can also be seen in the hippocampal content of NF-*κ*B p65. Results in Figure 4 show increased levels of NF-*κ*B p65 in STZ-treated mice and its gradual decrease after treatment with Antia.

**Figure 5:**
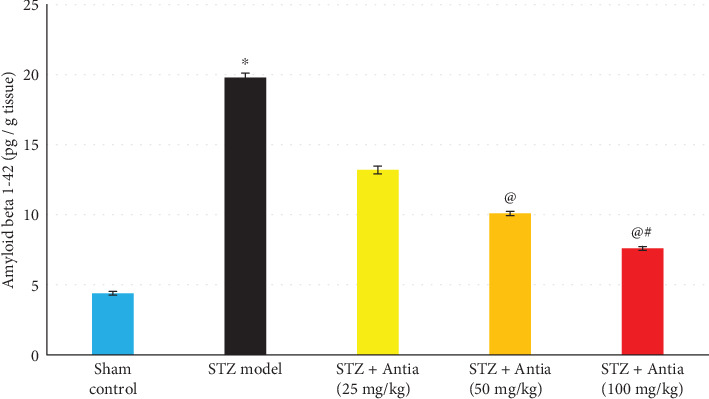
Effect of Antia on Amyloid *β*_1–42_ hippocampal content in ICV-STZ-injected mice. ^∗^Significantly different from the sham control group at *p* < 0.05. ^@^Significantly different from the ICV-STZ group at *p* < 0.05. ^#^Significantly different from Antia (25 mg/kg) at *p* < 0.05. ^$^Significantly different from Antia (50 mg/kg) at *p* < 0.05.

Since amyloid *β* makes up Alzheimer's disease plaques, we examined the effect of Antia on Amyloid *β*_1–42_ hippocampal content in ICV-STZ-injected mice. Results depicted in Figure 5 show that STZ model mice exhibited an approximately 4-fold increase in the expression of amyloid *β* as compared with sham control mice. However, amyloid *β* levels were significantly decreased after treatment with Antia. The effect was dose-dependent and reached its lowest levels at 100 mg/kg.

**Figure 6:**
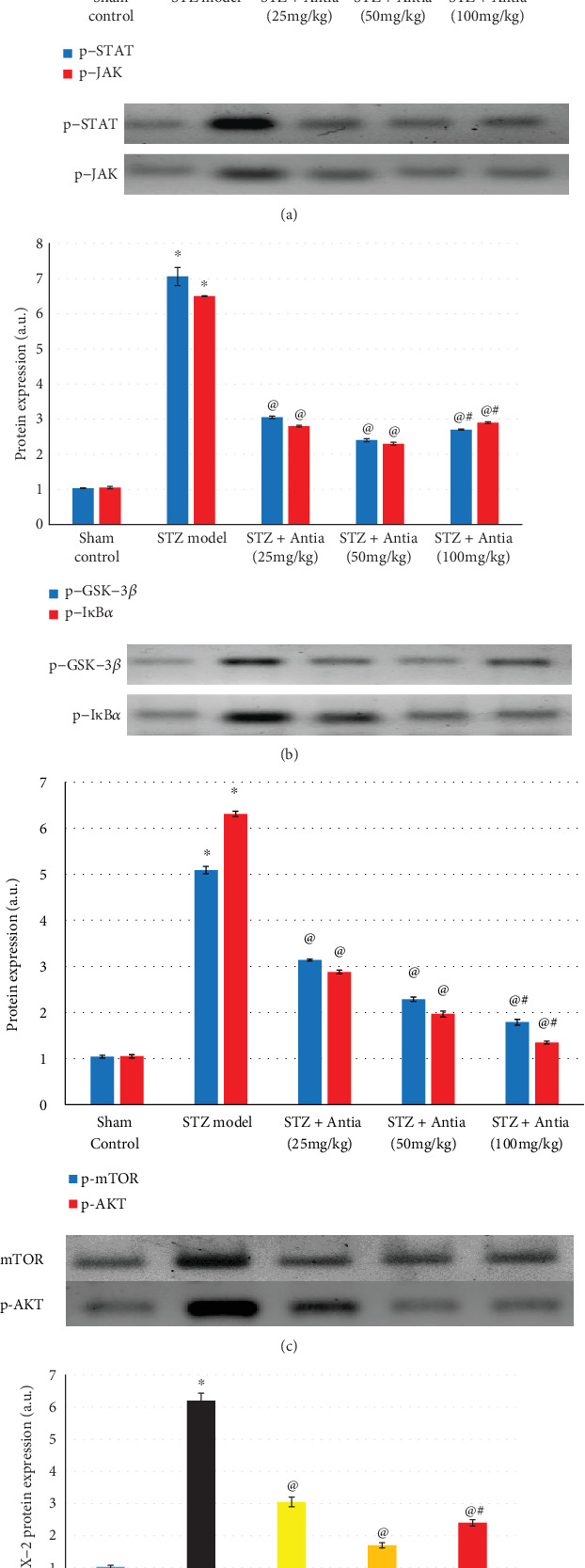
Effect of Antia on protein expression in the hippocampi of ICV-STZ-injected mice for (a) phosphorylated STAT and JAK, (b) GSK-3*β* and I*κ*B*α*, (c) mTOR and p-AKT, and (d) COX-2. ^∗^Significantly different from the sham control group at *p* < 0.05. ^@^Significantly different from the ICV-STZ group at *p* < 0.05. ^#^Significantly different from Antia (25 mg/kg) at *p* < 0.05. ^$^Significantly different from Antia (50 mg/kg) at *p* < 0.05.

We further examined protein expression. The levels of phosphorylation of STAT and JAK protein expression are a well-established method used in Alzheimer's research. We examined whether treatment with Antia suppresses the phosphorylation of STAT expression in STZ mice. As expected, the levels of phosphorylation of STAT protein expression were significantly increased when compared with sham control mice. However, treatment of STZ mice with Antia caused a significant inhibition in the phosphorylation level of STAT3 (Figure 6(a)). A similar trend in results was observed with JAK2 protein expression. Antia treatment resulted in a significant inhibition in the phosphorylation level of JAK2 due to of STZ injection (Figure 6(a)). These results indicate the protective effect of Antia for the JAK2/STAT3 pathway.

Earlier studies have shown that glycogen synthase kinase-3 (GSK-3) phosphorylates tau protein, the primary component of neurofibrillary tangles. GSK-3*β* inhibition presents a new method for reducing the formation of both neurofibrillary tangles and amyloid plaques, two of Alzheimer's disease's pathological hallmarks [47]. Results in Figure 6(b) show that STZ-treated mice had a higher expression of GSK-3*β* level that was 7-fold larger than the GSK-3*β* level of sham control mice. Antia treatment, however, resulted in a dramatic inhibition in the expression of GSK-3*β* that was approximately 3-fold of the control. Results in Figure 6(b) also show that STZ-treated mice had an approximately 6.5-fold larger I*κ*B*α* level expression than the sham control mice, whereas Antia treatment resulted in a dramatic inhibition in the expression of I*κ*B*α* that was approximately 2.8-fold of the control.

Several studies have shown that mTOR, rapamycin's mammalian target, may contribute to amyloid *β*- and tau-induced neurodegeneration [48]. Earlier studies showed that AD cases had higher levels than the control of mTOR phosphorylated at Ser2481 in the medial temporal cortex [49, 50]. Results in Figure 6(c) showed that STZ-injected mice exhibited significantly increased levels of the p-mTOR and p-AKT protein expression that were 5-6 fold greater than the level of sham control mice, respectively, but treatment with Antia reversed that increase and brought it close to that of the control values.

Finally, COX-2 is an important enzyme in inflammatory processes. Results in Figure 6(d) show that STZ-treated mice exhibited a significant induction in COX-2 expression of about 6-fold compared to sham control mice. Treatment with Antia, however, significantly reduced the expression of COX-2 to 150%-300%.

## 4. Discussion

The present study's results demonstrate that the antioxidant Antia exerts protective effects for mice induced with SAD. The constituents of Antia have previously been shown to possess various neuroregenerative and protective properties. Yamabushitake mushrooms have been shown to synthesize nerve growth factor [51–53]; gotsukora extracts reduce the amyloid *β* levels in the Alzheimer-stricken brains of laboratory animals [23]; diosgenin enhances the cognitive performance of mice [27]; amla acts as a potent antioxidant with strong neuroprotective effects and cognitive enhancement properties [28–31]; and kothala himbutu protects against deleterious cognitive changes in young diabetic rats [32] and against mercury toxicity in mice hippocampi [33]. Here, Antia is shown to attenuate cognitive dysfunction in the mouse model by targeting several linked pathways, including the amyloidogenic, inflammatory, autophagy, and oxidative stress pathways.

In the present study, induction of SAD in mice by STZ induced a significant cognitive decline in the Morris water maze and novel object recognition tests. ICV injection of STZ is an experimental model that mimics the progressive pathology of SAD similar to human brains [38]. STZ-treated mice showed significant memory and learning deficits, as shown by the mice's noticeable inability to discriminate between novel and familiar objects in the novel object recognition tasks and Morris water maze test. This is in harmony with previous studies [54, 55]. However, the profound elevation in escape latency during the acquisition trial and the time spent in the target quadrant during the probe trail in the Morris water maze test, as well as the increase in discrimination and preference indices in the novel object recognition test, proved that Antia prevented the STZ-induced impairments of spatial and short-term memory. This improvement in the object recognition memory deficit could be attributed to the previously proven effects of several of Antia's ingredients. For example, it has been shown that diosgenin has an antiamyloidogenic effect [27, 56] and that Hericium erinaceus has a strong neuroprotective effect against neuronal loss and dementia in AD [57, 58]. Furthermore, oral administration of dried yamabushitake mushroom powder has been demonstrated to effectively improve mild cognitive impairment in humans [19].

STZ administration significantly increased the hippocampal content expression of NF-*κ*B and anti-inflammatory cytokines, namely, IL-6 and TNF-*α*. NF-*κ*B plays a pivotal role in neurons' inflammatory responses by inducing the transcription of inflammatory target genes, including COX-2, TNF-*α*, IL-6, and IL-1*β* [59]. TNF-*α* is involved in systemic inflammation, and in particular, it is involved in AD-related brain neuroinflammation as well as amyloidogenesis via *β*-secretase regulation. Moreover, increased IL-6 expression in the brain is linked with the profound neuropathological changes found with Alzheimer's and Parkinson's disease [60]. NF-*κ*B has also been demonstrated to regulate the expression of BACE-1, the rate-limiting enzyme responsible for amyloid *β* production. The Janus Kinase/Signal Transducers and Activators of Transcription (JAK/STAT) signaling pathway emerged in the 1980s as the pathway mediating interferon signaling. Neuroinflammation is accompanied by diseases, and JAK2/STAT3 pathway activation leads to pathogenic inflammation. Thus, targeting the JAK2/STAT3 pathway can be used as a protective therapy for neuroinflammatory and neurodegenerative diseases like AD.

In the present study, administration of Antia was shown to have a significant anti-inflammatory effect, as demonstrated by decreasing the levels of all measured inflammatory cytokines as well as dramatically inhibiting the expression of phosphorylated STAT3 and JAK2. The STAT3/JAK2 pathway has been linked to TNF-*α* production [61, 62]. The significant inhibition of TNF-*α* and NF-*κ*B might be caused by the action of Hericium erinaceus, known as yamabushitake, which has been demonstrated to contribute to transcriptional regulation of adhesion molecules and numerous cytokines including TNF-*α* and IL-6 [63, 64].

Neuroinflammation has been linked to a deficit of autophagy, which may contribute to neurodegeneration [8]. mTOR, rapamycin's mammalian target, is known to regulate autophagy along with protein kinase B (Akt) [65]. Several studies emphasize the close link between cognitive impairment in AD and mTOR signaling and the presence of amyloid *β* plaques [66–69]. Furthermore, in human and rat studies of AD, autophagy activation has been linked to GSK-3*β* inhibitors and its deficit has been found to play a role in pathological tau aggregate accumulation [9, 11].

Treatment with Antia reversed the elevated expression of mTOR, Akt, I*κ*B*α*, and GSK-3*β* levels after STZ injection and brought it closer to that of the control. Recent reports found that increasing neurons' axonal density with diosgenin significantly improved cognitive function. This could be achieved through modulation of the PI3K-Akt pathway, a pathway that plays a key role in axon regeneration by regulating local protein translation via the mTOR pathway [27, 70]. Moreover, it was proven recently that amla, a major constituent of Antia, exerted a significant modulation of the p-AKT/GSK-3*β* pathway, resulting in a decline of phosphorylated tau and amelioration of cognitive deficits [71].

Results of this study showed that Antia increases GSH and decreases lipid peroxidation in STZ-treated mice. Previous research showed that the generation of ROS via amyloid *β* during self-aggregation may damage neurons and cause neuronal death [72]. Lipid peroxidation is considered to be a major outcome of injury mediated by free radicals that directly damages membranes, and increased lipid peroxidation has been found in AD patients' brains [73, 74]. Treatment of STZ-treated mice with Antia improved the oxidative stress parameters. This might be due to its previously known ability to reverse oxidative-stress-induced mitochondrial dysfunction and apoptosis [36]. In addition, Centella asiatica, commonly known as gotsukora, and MRN-100 have been found previously to provide multiple mechanisms for altering Alzheimer's brain pathology such as protection from DNA fragmentation due to oxidative stress, decreased lipid peroxidation, and the exhibition of noticeable free radical scavenging properties [23, 34]. GSH is an antioxidant that can prevent damage caused by ROS and may protect against oxidative and neurotoxic degeneration of oligomeric amyloid *β* [75, 76].

It could be concluded from the present study that Antia exerts a significant protection against sporadic AD induced by ICV injection of STZ. This effect is achieved through targeting the amyloidogenic, inflammatory, and oxidative stress pathways. The JAK2/STAT3 pathway played a protective role for the induced neuroinflammation, which is mediated through modulation of the Akt/mTOR/GSK-3*β* pathway. To our knowledge, this is the first work done to investigate the protective effect of Antia against neurodegenerative diseases such as SAD.

## Data Availability

The data of the present study including the figures and western blot analysis, used to support the findings of this study are included within the article.
